# TikTok and YouTube as sources of information on anal fissure: A comparative analysis

**DOI:** 10.3389/fpubh.2022.1000338

**Published:** 2022-11-03

**Authors:** Zeyang Chen, Shaorong Pan, Shuai Zuo

**Affiliations:** Department of General Surgery, Peking University First Hospital, Peking University, Beijing, China

**Keywords:** anal fissure, internet, quality, TikTok, YouTube

## Abstract

**Introduction:**

Anal fissure is a common colorectal disease impacting patients' life quality with high incidence. Social media platforms are becoming a kind of health information source nowadays. This study aims to evaluate and compare the quality of anal fissure-related videos on TikTok and YouTube.

**Materials and methods:**

One hundred videos were sourced from TikTok and YouTube, respectively and videos were screened further. The completeness of six types of content within the videos is assessed, including the definition of disease, symptoms, risk factors, evaluation, management and outcomes. Finally, the DISCERN instrument, Patient Education Materials Assessment Tool and Global Quality scale are used to assess video display quality and content. A correlation analysis is undertaken considering the video features, DISCERN, PEMAT and GQS scores.

**Results:**

Physicians and non-profit organizations contributed almost all video content among selected videos. A statistically significant correlation between DISCERN classification and duration, PEMAT understandability, PEMAT actionability and GQS scores is recorded. DISCERN total scores were significantly positively correlated with video duration, PEMAT understandability, PEMAT actionability and GQS scores. GQS scores were significantly positively correlated with duration, PEMAT understandability and PEMAT actionability scores. For content, the videos mainly described management and symptoms while containing limited information on the disease evaluation, and outcomes.

**Conclusions:**

The sources of uploaders on YouTube are more diverse than TikTok, and the quality of videos is also relatively higher on YouTube. Even so, the video quality of the two platforms still needs to be further improved. Health information without integrity, reliability and practicability impacts patients' disease perception and health-seeking behavior, leading to serious consequences. Much effort must be taken to improve the quality of videos regarding anal fissures on the two platforms, which will facilitate the development of public health education on this issue.

## Introduction

Anal fissures are one of the most common proctology diseases, having a high morbidity rate and causing overwhelming pain in patients (1). People between 30 and 50 years old are considered the vulnerable population to anal fissures, without a significant difference in incidence rates between men and women (2). The longitudinal defect located at the anoderm between the anal verge and dentate line can cause severe pain during defecation and may persist for several hours (3). Although some debate exists on the exact pathogenesis, dry and hard stools may correlate with the occurrence of anal fissures (4). An acute anal fissure with a course of < 6 weeks has the possibility of recovering and healing when conservative treatment is used (2, 5). Without sufficient attention and timely treatment, an acute anal fissure can become chronic and may need operative treatment (6). Patients with chronic anal fissures always have a poor quality of life and sexual function (2). Surgical procedures, such as lateral internal sphincterotomy, incur some risk and can lead to fecal incontinence (7).

As a short-video app, TikTok has increasingly attracted researchers focused on its potential to facilitate health communications (8–10), the same for YouTube (11–16). Due to the rich technological functions of them, such as yielding likes, comments, chat, and live streaming, they has been regarded as a reliable source of health information with favorable public acceptance. The coronavirus-related videos on TikTok were watched nearly 93.1 billion times during the COVID-19 pandemic in July 2020 (17). The advancing internet technologies have transformed patients from passive healthy information receivers to active consumers. Since the global spread of COVID-19, the sharply increased number of critical patients and over-constricted medical resources may make timely diagnosis and treatment of anal fissures challenging. The internet has the potential to become an ideal source for patients with anal fissures to source scientific and medical knowledge. Almost half of the patients with an acute anal fissure can be successfully treated using conservative treatments (5). The timely application of these simple non-operative treatment methods, which can be undertaken at home, such as sitz baths and fiber supplementation, would be vital in preventing patients from undergoing surgical treatment. The two platforms have considerable potential to improve the prognosis of anal fissure patients.

Some scholars have previously evaluated the information quality of some common diseases featured on TikTok and YouTube, such as bladder cancer, colorectal cancer, diabetes and chronic obstructive pulmonary diseases (8, 9, 12, 13). This study aims to evaluate and compare the quality of videos describing anal fissures on TikTok and YouTube so that provide some fact-based recommendations for better public health engagement.

## Materials and methods

### Search strategy and data extraction

We employed the search term “肛裂” (“anal fissure” in Chinese) on TikTok (known as Douyin in mainland China) and “Anal fissure” on YouTube to retrieve the related videos on May 30th, 2022. Before undertaking the search, the search history was deleted to reduce the influence of previous searches on the search results and outcomes. The top 100 videos extracted according to the default search mode were selected.

**Figure 1:**
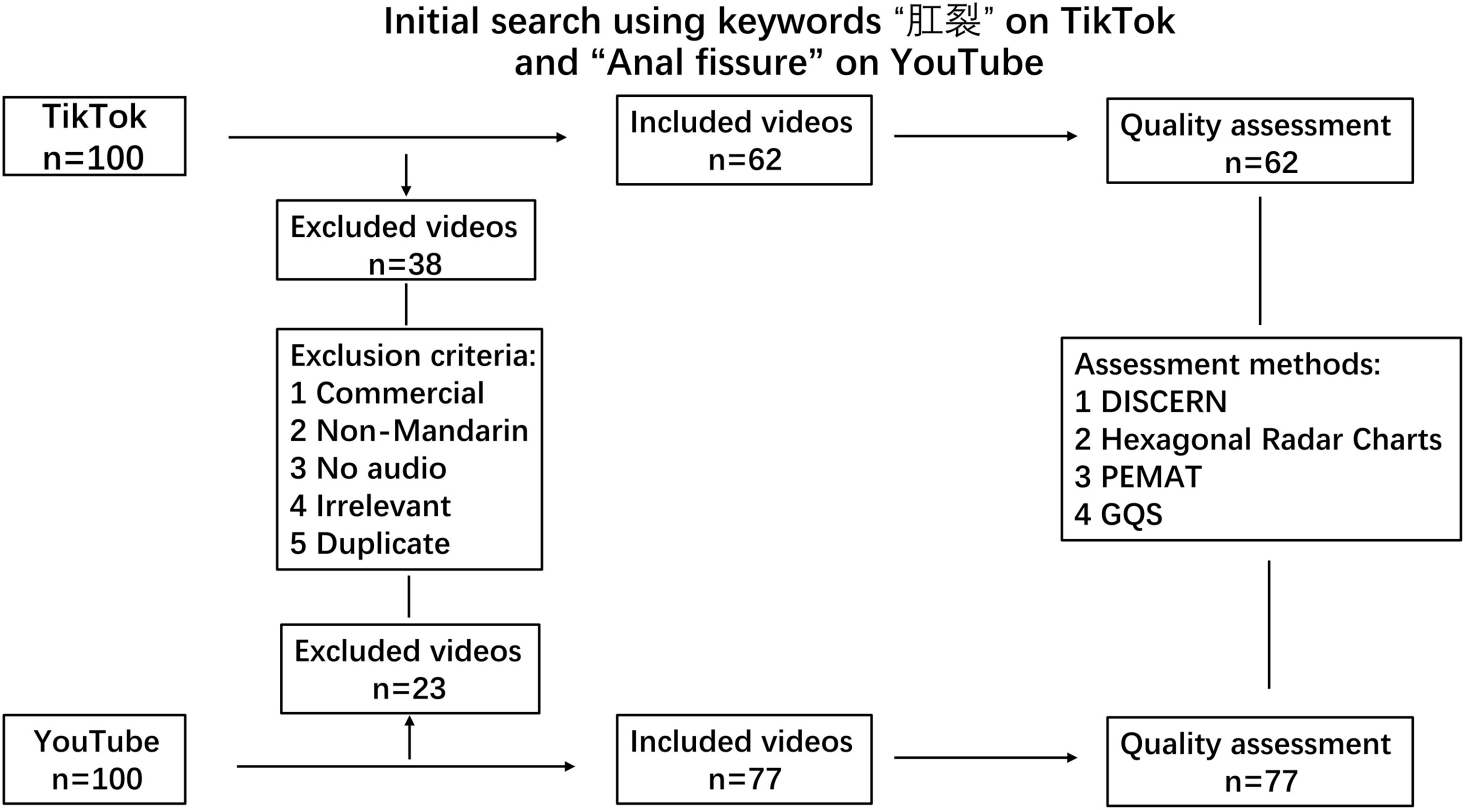
Flowchart of the selection of videos included in the analysis.

To screen the most relevant videos, videos were excluded according to the following criteria: (1) commercial; (2) non-Mandarin; (3) no audio; (4) irrelevant; and (5) duplicate. Following active filtering, 62 videos on TikTok and 77 videos on YouTube remained for further data extraction and quality assessment (see details in Figure 1).

The basic information of each video considered and extracted included the URL, upload day, type of uploader, duration of the video and number of views, likes, comments, and collects it received. Excel (Microsoft Inc) spreadsheets were used to record, collate and analyse the extracted data.

### Assessment procedure

The sample videos were analyzed in two aspects: the video content and the quality of video information.

The video content was assessed by considering six dimensions, including the definition of the disease, symptoms, risk factors, evaluation, management and outcomes, which were visually represented with the Hexagonal Radar Chart (18). Each dimension was scored on a 5-item scale: 0 points (no content), 0.5 points (little content), 1 point (some content), 1.5 points (most content), and 2 points (extensive content).

The DISCERN instrument, Patient Education Materials Assessment Tool (PEMAT) and Global Quality scale (GQS) were used to assess the quality of the video information.

DISCERN is designed as a brief questionnaire to assist users in scoring the quality of health information (19). It has been one of the most widely used tools for assessing the quality of health information since its publication (20). DISCERN contains three sections, the reliability of publication (8 items), the quality of information on treatment choices (7 items) and the overall score of the publication (1 item). The 16 questions of the DISCERN scale are rated on a scale of 1 (poor) to 5 (good). The total DISCERN score is calculated by summing the scores over all 16 questions. All videos were divided into five categories according to the total DISCERN score: very poor (< 27), poor (27–38), fair (38–50), good (51–62), and excellent (63–80). Notably, although DISCERN was initially designed for assessing written publications, it has been widely applied for evaluating videos related to health (21). The complete questionnaire is presented in Supplementary Table S1.

The videos were evaluated for understandability and actionability using the Patient Education Materials Assessment Tool (PEMAT) (22). The PEMAT consists of 17 items and two subscales. Thirteen items are related to understandability, and four are relevant to actionability. Each item is rated as agree (1 point), disagree (0 points) or not applicable (no point and noted as not applicable). Final scores are calculated as a percentage of agreed responses for all items, excluding those scored as not applicable. Higher percentages indicate higher understandability or actionability. Scores above 70% indicate that the information is easily understood or actionable.

GQS is a 5-point scale (1–5) instrument that measures the flow, quality and usefulness of the video, which was also used for the quality analysis. One or two points indicate low quality, three medium quality, and 4 or 5 high quality (23). The detailed description of each point is presented in Supplementary Table S2.

Two independent raters (CZY and PSR) assessed all videos. Discussion with a third author (ZS) is necessary when discrepancies arise between reviewers, to receive consensus.

### Statistical analysis

SPSS software version 26.0 (SPSS Inc., Chicago, IL, USA) was used for all statistical analyses. Categorical variables were presented as frequency and ratios (%), and continuous variables were presented by mean ± standard deviation (SD). The Kruskal-Wallis test was used to determine statistically significant differences involving more than two groups of any independent variable. Among variables, Spearman's correlation coefficient was employed to assess any correlations. Statistically significant relationships were identified when the *P*-value < 0.05.

### Ethics approval

This study focused on the quality assessment of TikTok and YouTube videos contributed and viewed by the public, so ethics committee approval was unnecessary.

## Results

### Video characteristics

The anal fissure-related videos on TikTok mainly arise from two source types: physicians and non-profit organizations. The physicians contributed almost all videos in the study sample (61/62, 98.38%), while quite a small number of the videos are contributed by non-profit organizations (1/62, 1.61%). However, the video sources on YouTube are relatively diverse, they also include another two source types: normal user and profit organization. The two sources make up a small part, 2.60% (2/77) and 5.19% (4/77), respectively. The mean length of the videos considered on TikTok was 39.26 seconds, varying from 16.00 to 184.00 seconds, and it was up to 600.06 seconds, ranging from 28.00 to 5636.00 seconds on YouTube. The most recent video was uploaded 33 days on TikTok and 1 day on YouTube before data collection, whereas the oldest one had been on the two platforms for over 2 years. Even though this, our results also show that there exists statistically significantly different between the two platforms on video duration and online days. The number of likes ranged from 53 to 106000 for each video on TikTok, and the number of comments and collects ranged from 0 to 6924, and 3–5150, respectively. As for YouTube, the number of views, likes and comments varied from 30–1511139, 0–28000 and 0–2095, respectively. More detailed information about each video's features is shown in Table 1.

**Table 1:** General features of included videos.

	**TikTok**		**YouTube**		***P*-value^a^**
**Source of upload**	**N**	**%**	**N**	**%**	**–**
Physician	61	98.38	30	38.96	–
Normal user	–	–	2	2.60	–
Non–profit organization	1	1.61	41	53.25	–
Profit organization	–	–	4	5.19	–
**Video features**	**Mean** ±**Std. Deviation**	**Min – Max**	**Mean** ±**Std. Deviation**	**Min – Max**	**–**
Duration(s)	39.26 ± 27.62	16.00–184.00	600.06 ± 798.26	28.00–5636.00	**< 0.001**
Number of days online	263.65 ± 182.15	33.00–876.00	902.01 ± 929.72	1.00–3715.00	**< 0.001**
Number of views	–	–	139791.62 ± 302294.67	30.00–1511139.00	
Number of views/day	–	–	247.81 ± 801.07	0.07–6476.44	
Number of likes	5340.53 ± 15737.6	53.00–106000.00	1317.70 ± 3670.77	0–28000.00	0.053
Number of likes/day	27.33 ± 68.99	0.10–366.20	4.17 ± 19.70	0–167.22	**0.013**
Number of comments	594.45 ± 1421.24	0–6924.00	171.42 ± 370.02	0–2095.00	**0.026**
Number of comments/day	4.31 ± 14.20	0–97.52	0.54 ± 2.38	0–20.56	**0.043**
Number of collects	288.16 ± 778.59	3.00–5150.00	–	–	
Number of collects/day	1.46 ± 3.16	0.01–18.51	–	–	
DISCERN quality	2.56 ± 1.10	1–4.80	2.45 ± 1.11	1.00–5.00	0.59
DISCERN reliability	17.63 ± 3.60	10.50–23.20	21.88 ± 5.85	8.00–40.00	**< 0.001**
DISCERN treatment	13.68 ± 5.82	7.00–27.40	15.82 ± 6.70	7.00–29.00	0.05
DISCERN total	33.86 ± 9.10	19.20–55.10	40.16 ± 12.51	16.00–68.00	**0.001**
PEMAT understandability total points	6.98 ± 1.03	4.00–9.00	8.14 ± 2.62	2.00–13.00	**0.001**
PEMAT understandability total possible points	9.08 ± 0.27	9.00–10.00	9.62 ± 2.51	6.00–13.00	0.064
PEMAT understandability score (%)	76.86 ± 10.83	44.44–100.00	85.31 ± 16.21	16.67–100.00	**< 0.001**
PEMAT actionability total points	1.35 ± 1.04	0–3.00	1.74 ± 0.92	0–4.00	**0.024**
PEMAT actionability total possible points	3.00 ± 0	3.00–3.00	3.03 ± 0.16	3.00–4.00	0.159
PEMAT actionability score (%)	45.16 ± 34.72	0–100.00	57.14 ± 29.05	0–100.00	**0.032**
GQS score	2.93 ± 0.92	1.00–4.60	2.92 ± 0.89	2.00–5.00	0.956

a *t*–test. The bold values indicate the *p*-value less than 0.05.

### Video quality and content

The mean DISCERN total score, PEMAT understandability score, PEMAT actionability score and GQS score for TikTok is 33.86 (range 19.2–55.1), 76.86% (range 44.44–100%), 45.16% (range 0–100%) and 2.93 (range 1–4.6), respectively. Regarding YouTube, they are 40.16 (range 16.00–68.00), 85.31% (range 16.67–100%), 57.14% (range 0–100%) and 2.92 (range 2.00–5.00), respectively. Besides, we also found that the DISCERN total score, PEMAT understandability score, PEMAT actionability score of YouTube were statistically significantly higher than these of TikTok (see Table 1).

The DISCERN classification scores for TikTok are 19.4% were “very poor”, 51.6% were “poor”, 25.8% were “fair,” 3.2% were “good” and none were “excellent”. For YouTube, 16.9% were “very poor”, 41.6% were “poor”, 35.1% were “fair,” 20.8% were “good” and 2.6% were “excellent”. The results showed that there was a statistically significant correlation between DISCERN classification and video duration, PEMAT understandability score, PEMAT actionability score and GQS score on the both platforms. Besides, the DISCERN classification was also statistically significant correlated with likes/day and comments/day on YouTube (see Table 2).

**Table 2:** Distribution of DISCERN classification according to the video features.

**Variable**		**Very poor**	**Poor**	**Fair**	**Good**	**Excellent**	***P*-value^a^**
Number of videos	TikTok	12 (19.4%)	32 (51.6%)	16 (25.8%)	2 (3.2%)	0	–
	YouTube	13 (16.9%)	19 (24.7%)	27 (35.1%)	16 (20.8%)	2 (2.6%)	–
Duration(s)	TikTok	27.83 ± 8.56	33.84 ± 15.63	55.25 ± 44.08	66.50 ± 30.41	–	**0.036**
	YouTube	386.38 ± 530.95	372.53 ± 449.74	562.41 ± 487.08	1123.44 ± 1393.59	472.00 ± 272.94	**0.015**
Number of views	TikTok	–	–	–	–	–	–
	YouTube	285954.62 ± 493656.01	56306.37 ± 97450.63	67036.15 ± 104663.93	162332.56 ± 272822.93	784713.50 ± 1027320.79	0.298
Number of views/day	TikTok	–	–	–	–	–	–
	YouTube	255.94 ± 521.37	86.95 ± 164.43	91.90 ± 203.83	254.09 ± 469.43	3777.53 ± 3816.84	0.054
Number of likes	TikTok	9477.67 ± 15596.50	2709.13 ± 7178.00	1520.81 ± 1077.79	53178.00 ± 74701.59	–	0.235
	YouTube	1472.77 ± 3576.96	505.74 ± 885.84	536.07 ± 858.47	2940.81 ± 6811.07	5590.50 ± 5777.77	0.115
Likes/day	TikTok	64.13 ± 117.57	17.79 ± 38.37	4.59 ± 2.97	141.11 ± 198.07	–	0.559
	YouTube	1.49 ± 3.28	0.90 ± 1.45	0.96 ± 1.61	5.31 ± 11.79	87.06 ± 113.36	**0.039**
Number of comments	TikTok	1527.08 ± 2615.37	446.97 ± 1038.85	260.00 ± 253.79	34.00 ± 4.24	–	0.206
	YouTube	113.62 ± 268.71	77.84 ± 121.31	130.44 ± 222.59	277.56 ± 506.63	1140.00 ± 1350.57	0.075
Comments/day	TikTok	12.56 ± 28.52	3.26 ± 8.54	0.73 ± 0.66	0.10 ± 0.02	–	0.357
	YouTube	0.12 ± 0.25	0.17 ± 0.26	0.20 ± 0.28	0.59 ± 1.02	11.03 ± 13.48	**0.02**
Number of collects	TikTok	362.33 ± 503.08	272.91 ± 907.26	103.69 ± 87.90	1563 ± 2166.58	–	0.594
	YouTube	–	–	–	–	–	–
Collects/day	TikTok	2.75 ± 5.43	1.34 ± 2.51	0.38 ± 0.4	4.15 ± 5.74	–	0.551
	YouTube	–	–	–	–	–	–
PEMAT understandability score (%)	TikTok	67.59 ± 8.81	78.37 ± 8.75	81.39 ± 12.62	72.22 ± 7.86	–	**0.002**
	YouTube	75.06 ± 23.28	82.35 ± 17.63	86.70 ± 12.23	92.97 ± 8.29	100.00 ± 0.00	**0.024**
PEMAT actionability score (%)	TikTok	22.22 ± 32.82	39.58 ± 32.17	66.67 ± 24.34	100.00 ± 0.00	–	**0.001**
	YouTube	25.64 ± 33.76	47.37 ± 27.92	64.20 ± 15.81	77.08 ± 15.96	100.00 ± 0.00	**< 0.001**
GQS score	TikTok	1.70 ± 0.34	2.84 ± 0.55	3.85 ± 0.58	4.40 ± 0.14	–	**< 0.001**
	YouTube	2.00 ± 0.00	2.26 ± 0.45	3.19 ± 0.62	3.75 ± 0.58	5.00 ± 0.00	**< 0.001**

a *Kruskal*–*Wallis* test. The bold values indicate the *p*-value less than 0.05.

The correlation test indicated that DISCERN total scores were significantly positively correlated with video duration, PEMAT understandability score, PEMAT actionability score and GQS score on the two platforms. Meanwhile, the GQS scores were significantly positively correlated with duration, PEMAT understandability score, and PEMAT actionability score on TikTok and YouTube. Moreover, the DISCERN total scores and GQS scores were also significantly positively correlated with likes/day and comments/day on YouTube, and number of comments was significantly positively correlated with the GQS scores on YouTube, too (see Table 3).

**Table 3:** Correlation analyses for DISCERN score and GQS score.

	**TikTok**				**YouTube**			
	**DISCERN**		**GQS**		**DISCERN**		**GQS**	
	**r**	***P*-value^a^**	**r**	***P*-value^a^**	**r**	***P*-value^a^**	**r**	***P*-value^a^**
DISCERN	–	–	0.830	**< 0.001**	–	–	0.838	**< 0.001**
GQS	0.830	**< 0.001**	–	–	0.838	**< 0.001**	–	–
Duration(s)	0.476	**< 0.001**	0.406	**< 0.001**	0.428	**< 0.001**	0.451	**< 0.001**
Number of likes	0.046	0.721	−0.002	0.988	0.180	0.117	0.195	0.090
Likes/day	−0.090	0.486	−0.167	0.194	0.257	**0.024**	0.266	**0.02**
Number of comments	0.031	0.810	−0.055	0.670	0.211	0.065	0.241	**0.034**
Comments/day	−0.061	0.639	−0.165	0.199	0.285	**0.012**	0.313	**0.006**
PEMAT understandability score (%)	0.466	**< 0.001**	0.473	**< 0.001**	0.323	**0.004**	0.380	**0.001**
PEMAT actionability score (%)	0.647	**< 0.001**	0.467	**< 0.001**	0.639	**< 0.001**	0.651	**< 0.001**

a *Spearman* test. The bold values indicate the *p*-value less than 0.05.

**Figure 2:**
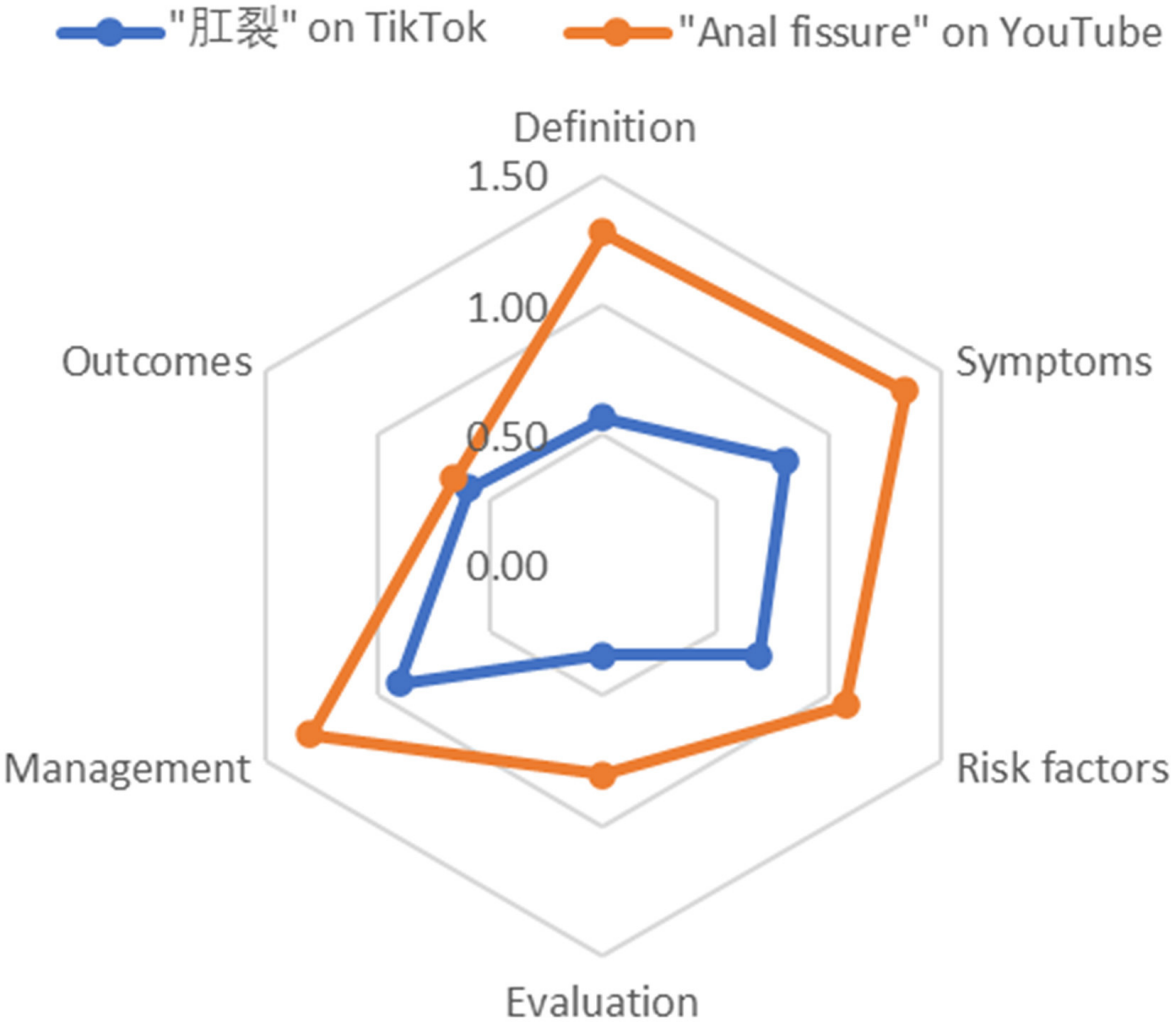
Completeness of video content.

The Hexagonal Radar Chart illustrates the imbalanced content of information on TikTok and YouTube. The six dimensions were discussed more on YouTube than TikTok. The mean scores of six dimensions indicated that the management and symptoms of anal fissure were discussed more thoroughly compared to the definition of the disease, risk factors, evaluation, and outcomes on the both platforms, although all dimensions have an average score of no more than 1 point on TikTok. Detailed mean scores and the Hexagonal Radar Charts are presented in Table 4 and Figure 2.

**Table 4:** Completeness of video content.

**Item**	**Definition**	**Symptoms**	**Risk factors**	**Evaluation**	**Management**	**Outcomes**
“肛裂” on TikTok	0.57	0.81	0.69	0.35	0.91	0.60
“Anal fissure” on YouTube	1.28	1.34	1.07	0.81	1.30	0.66

## Discussion

In this era of rapid scientific and technological developments, previous studies have focused on evaluating the quality of videos about colorectal diseases, including benign (14) and malignant diseases (13, 16), on social media platforms. Colorectal diseases not only include malignancies, such as colorectal carcinoma, but also include benign diseases, such as hemorrhoids, anal fistulas, and anal fissures. However, as a benign disease with high incidence, anal fissures have not attracted investigation, and the role of social media in disseminating medical information about this disease remains unclear. TikTok is one of the most popular short-video social media platforms in China, while YouTube is another widely used social media all over the world, and some studies have addressed their enormous potential in popularizing science about medicine (8–13). Statistical analyses revealed that 62 videos on TikTok and 77 videos on YouTube selected in this study had received 331113 and 101463 likes, 36856 and 13199 comments respectively, which is smaller than other studies concerning some common chronic diseases, such as diabetes (8) and chronic obstructive pulmonary disease (9). The dissatisfactory result makes our study, measuring the quality of existing videos on TikTok and YouTube with a high penetration rate, more meaningful. The outcomes from this study may help improve the quality of videos about health and medical information about anal fissures on social media, attracting more people to be aware of this common and painful anal disease, potentially resulting in positive effects on public health promotion.

Some studies themed by videos about malignant tumors have a vast range of information sources, such as TV programs, individual users, and health information websites (12, 24). Unlike these studies, almost all of the uploaders of videos on TikTok in our study are physicians. In contrast to life-threatening diseases, only health professionals value the publicity of health and medical information about anal fissures on TikTok. The uploaded sources of anal fissure videos on YouTube are more diverse than TikTok. Even though the sources of videos on YouTube are more extensive, they are mainly physicians and non-profit organizations, and videos from other sources still account for only a very small part. This outcome infers anal fissures are accorded insufficient social and public attention.

Various assessment instruments, with different emphases, are applied to assess the quality of the selected videos. The DISCERN scale focuses more on integrity and reliability, PEMAT understandability and actionability, with GQS considering flow and usefulness. The Hexagonal Radar Chart measures the content of videos in multiple dimensions and reflects whether they are comprehensive and complete. The mean DISCERN and GQS scores for TikTok and YouTube are 33.86 and 2.93, 40.16 and 2.92, respectively. According to the criterion of DISCERN classification, 71% (44/62) of videos were of very poor or poor quality, and only 3.2% (2/62) videos were evaluated as having good quality on TikTok. However, regard to YouTube, 23.4% (18/77) videos were of good or excellent quality, and only 41.6% (32/77) were measured as very poor or poor quality. We also found that the duration, online time, and number of comments of videos on YouTube are statistically higher than those on TikTok, and the DISCERN total score, PEMAT understandability score, PEMAT actionability score of YouTube were statistically significantly higher than these of TikTok too. This may be related to the longer video duration on YouTube, which brings more useful information. This requires the TikTok platform to release the limit on the duration of uploaded videos as much as possible to achieve the purpose of delivering more useful information to users in one single video. We can conclude the quality of annal fissure videos on YouTube is relatively higher than TikTok. Even though this, the low DISCERN and GQS scores still revealed that the videos about anal fissures on the both platforms were of poor integrity, reliability, and practicability.

Mesut et al. (24) suggested that videos with long duration had better quality, which was consistent with this study. TikTok is an app that prioritizes uploading videos with a short duration. The shorter duration compared with YouTube may make it difficult for TikTok content to describe a disease clearly. This could be attributed to the professionalism of the video's creators, who are predominantly physicians, that the mean PEMAT understandability score was tolerableness (76.86%). However, the mean PEMAT actionability score was barely satisfactory. Uploaders of videos always divide the vital information about anal fissures into several sections, such as definition, symptoms, causes of sphincterismus, conservative treatment, and surgical treatment, which results in achieving more likes and comments. Notably, media platforms always recommend videos to users based on specific algorithms and randomness (25). Hence, the fragmented knowledge points mean that patients with anal fissures cannot get enough useful information from videos to sufficiently complete the self-care. The Hexagonal Radar Chart revealed that the symptoms and management of anal fissures was described more. The possible explanation may be that most videos target laypeople, who pay more attention to disease management rather than evaluation (9). However, scores of all aspects in the radar chart were lower than one, indicating partial and low-grade contents of videos on TikTok from another viewpoint. On the contrary, the videos on YouTube have relatively higher-grade contents on the whole.

The quality of videos does not necessarily match well with the popularity among the users (11, 24). The current study concludes that high-quality videos do not always have more likes, shares, and collects than those with lower quality. Keeland et al. (15) discovered that a surprising proportion of videos opposing immunization had received more views than supporting the immunization. Strangely enough, supporting videos were consistent with standard references instead of opposed videos. Some studies also advised that credibility depended on viewers' perception, which probably could not reflect the quality of videos objectively (26). This conclusion reinforces videos with incorrect information and low quality may be popular among people, influencing the patients' cognition about the disease and leading to dangerous consequences.

During the COVID-19 pandemic, because of the features of non-contact and convenience, the effect of social media on the promotion of public health has become increasingly important (27). Especially TikTok, a short-videos platform with more than 250 million active users on Chinese mainland, and YouTube, a widely used video platform with more than 2 billion users all over the world, show enormous value in science's popularization (28). The depressing results of the current study ring alarm bells. Concrete guidelines and measures are urgently required to improve the quality of videos with health, medical and scientific information. Firstly, the platforms need to encourage everyone who uploads videos, not only health professionals and non-profit organizations, to produce high-class videos about diseases, especially the common benign disease such as anal fissures. The physicians also should constrain themselves, from ethical and legal perspectives, to promote public health education and knowledge using their specialized training instead of enabling the apps as an instrument of self-promotion. Simultaneously, social media platforms desiderate the participation of physicians in different fields to assist in maintaining a high quality of videos. Take the acute anal fissure, for example, where patients receive correct and timely management advice such as sitz baths and fiber supplementation. These patients can self-manage at home, and may avoid surgical treatments due to the development of a chronic anal fissure. Finally, the algorithms of platform should consider the quality of videos and recommend high-quality videos as a priority.

This study has several limitations. Firstly, observer bias is inevitable due to our research's subjective evaluation instruments. Secondly, our study is a cross-sectional analysis that merely reflects video quality at a single time point. The outcomes may change over time because the selected videos may change when searching the index term. Finally, only one search term was used, “anal fissure”, and the results may be different if more terms had been chosen.

## Conclusion

This research is the first report to evaluate the quality of videos about anal fissures on social media platforms worldwide. As one of the most popular social media platforms, TikTok provides viewers with videos about anal fissures of poor quality, even if most uploaders are physicians. The sources of uploaders on YouTube are more diverse than TikTok, and the quality of videos is also relatively higher on YouTube. Even so, the video quality of the two platforms still needs to be further improved. Without accurate and comprehensive health education, patients may ignore the severity of the disease and then delay the diagnosis and treatment. It is vital to enhance the collaboration between social media and health professionals to improve the videos' quality of describing anal fissures, enabling and facilitating patient self-education.

## Data availability statement

The original contributions presented in the study are included in the article/Supplementary material, further inquiries can be directed to the corresponding author.

## Author contributions

ZC: acquisition of data and drafting of manuscript. SP: acquisition of data and design of statistical methods. SZ: critical revision of the manuscript for important intellectual content and drafting of manuscript. All authors contributed to the article and approved the submitted version.

## Funding

This work was supported by Youth Clinical Research Project of Peking University First Hospital (Grant No. 2017CR19).

## Conflict of interest

The authors declare that the research was conducted in the absence of any commercial or financial relationships that could be construed as a potential conflict of interest.

## Publisher's note

All claims expressed in this article are solely those of the authors and do not necessarily represent those of their affiliated organizations, or those of the publisher, the editors and the reviewers. Any product that may be evaluated in this article, or claim that may be made by its manufacturer, is not guaranteed or endorsed by the publisher.

## References

[1] van Reijn-Baggen DA Elzevier HW Putter H Pelger RCM Han-Geurts IJM. Pelvic floor physical therapy in patients with chronic anal fissure: a randomized controlled trial. Tech Coloproctol. (2022) 26:571–82. 10.1007/s10151-022-02618-9 35511322 PMC9069957

[2] Navarro-Sánchez A Luri-Prieto P Compañ-Rosique A Navarro-Ortiz R Berenguer-Soler M Gil-Guillén VF. Sexuality, quality of life, anxiety, depression, and anger in patients with anal fissure. A case-control study. J Clin Med. (2021) 10:4401. 10.3390/jcm10194401 34640419 PMC8509279

[3] Wald A Bharucha AE Limketkai B Malcolm A Remes-Troche JM Whitehead WE. Clinical guidelines: management of benign anorectal disorders. Am J Gastroenterol. (2021) 116:1987–2008. 10.14309/ajg.0000000000001507 34618700

[4] Cotton MH. Aetiology and treatment of anal fissure. Br J Surg. (1997) 84:279. 10.1002/bjs.1800840241 9052457

[5] Stewart DB Gaertner W Glasgow S Migaly J Feingold D Steele SR. Clinical practice guideline for the management of anal fissures. Dis Colon Rectum. (2017) 60:7–14. 10.1097/DCR.0000000000000735 27926552

[6] Luri-Prieto P Candela-Gomis A Palazón-Bru A Navarro-Cremades F Gil-Guillén VF Compañ-Rosique AF. Impact of anal fissure on neuroticism, extraversion, openness to experience, agreeableness, and conscientiousness: a case-control study. Visc Med. (2021) 37:128–33. 10.1159/000507382 33981753 PMC8077484

[7] Garg P Garg M Menon GR. Long-term continence disturbance after lateral internal sphincterotomy for chronic anal fissure: a systematic review and meta-analysis. Colorectal Dis. (2013) 15:e104–117. 10.1111/codi.12108 23320551

[8] Kong W Song S Zhao YC Zhu Q Sha L. TikTok as a health information source: assessment of the quality of information in diabetes-related videos. J Med Internet Res. (2021) 23:e30409. 10.2196/30409 34468327 PMC8444042

[9] Song S Xue X Zhao YC Li J Zhu Q Zhao M. Short-video apps as a health information source for chronic obstructive pulmonary disease: information quality assessment of TikTok videos. J Med Internet Res. (2021) 23:e28318. 10.2196/28318 34931996 PMC8726035

[10] Xue X Yang X Xu W Liu G Xie Y Ji Z. TikTok as an information hodgepodge: evaluation of the quality and reliability of genitourinary cancers related content. Front Oncol. (2022) 12:789956. 10.3389/fonc.2022.789956 35242704 PMC8885733

[11] Bai G Pan X Zhao T Chen X Liu G Fu W. Quality assessment of YouTube videos as an information source for testicular torsion. Frontiers in public health. (2022) 10:905609. 10.3389/fpubh.2022.905609 35664123 PMC9157819

[12] Loeb S Reines K Abu-Salha Y French W Butaney M Macaluso JN. Quality of bladder cancer information on YouTube. Eur Urol. (2021) 79:56–9. 10.1016/j.eururo.2020.09.014 33010986

[13] Joob B Wiwanitkit V. YouTube videos as a source of information on colorectal cancer: problem of the correctness of the contents. J Cancer Edu Official J Am Associat Cancer Edu. (2021) 36:652. 10.1007/s13187-020-01818-x 32617908

[14] Sturiale A Dowais R Porzio FC Brusciano L Gallo G Morganti R. YouTube as a source of patients' and specialists' information on hemorrhoids and hemorrhoid surgery. Rev Recent Clin Trials. (2020) 15:219–26. 10.2174/1574887115666200525001619 32448106

[15] Keelan J Pavri-Garcia V Tomlinson G Wilson K. YouTube as a source of information on immunization: a content analysis. JAMA. (2007) 298:2482–4. 10.1001/jama.298.21.2482 18056901

[16] Brar J Ferdous M Abedin T Turin TC. Online information for colorectal cancer screening: a content analysis of YouTube videos. J Cancer Edu Official J Am Assoc Cancer Edu. (2021) 36:826–31. 10.1007/s13187-020-01710-8 32072485

[17] Ostrovsky AM Chen JR. TikTok and its role in COVID-19 information propagation. J Adolescent Health Official Publ Soc Adolescent Med. (2020) 67:730. 10.1016/j.jadohealth.2020.07.039 32873499 PMC7455791

[18] Goobie GC Guler SA Johannson KA Fisher JH Ryerson CJ. YouTube videos as a source of misinformation on idiopathic pulmonary fibrosis. Ann Am Thorac Soc. (2019) 16:572–9. 10.1513/AnnalsATS.201809-644OC 30608877

[19] Charnock D Shepperd S Needham G Gann R. DISCERN an instrument for judging the quality of written consumer health information on treatment choices. J Epidemiol Commun Health. (1999) 53:105–11. 10.1136/jech.53.2.105 10396471 PMC1756830

[20] Zhang Y Sun Y Xie B. Quality of health information for consumers on the web: a systematic review of indicators, criteria, tools, evaluation results. J Assoc Inf Sci Technol. (2015) 66:2071–84. 10.1002/asi.23311

[21] Aydin MF Aydin MA. Quality and reliability of information available on YouTube and Google pertaining gastroesophageal reflux disease. Int J Med Inform. (2020) 137:104107. 10.1016/j.ijmedinf.2020.104107 32146372

[22] Shoemaker SJ Wolf MS Brach C. Development of the patient education materials assessment tool (PEMAT): a new measure of understandability and actionability for print and audiovisual patient information. Patient Educ Couns. (2014) 96:395–403. 10.1016/j.pec.2014.05.027 24973195 PMC5085258

[23] Bernard A Langille M Hughes S Rose C Leddin D Veldhuyzen van Zanten S. A systematic review of patient inflammatory bowel disease information resources on the World Wide Web. Am J Gastroenterol. (2007) 102:2070–7. 10.1111/j.1572-0241.2007.01325.x 17511753

[24] Duran MB Kizilkan Y. Quality analysis of testicular cancer videos on YouTube. Andrologia. (2021) 53:e14118. 10.1111/and.14118 34009641

[25] Sá SL Rocha AAA Paes A. Predicting popularity of video streaming services with representation learning: a survey and a real-world case study. Sensors. (2021) 21:7328. 10.3390/s21217328 34770633 PMC8588537

[26] Song S Zhang Y Yu B. Interventions to support consumer evaluation of online health information credibility: a scoping review. Int J Med Inform. (2021) 145:104321. 10.1016/j.ijmedinf.2020.104321 33202372

[27] Gunasekeran DV Chew A Chandrasekar EK Rajendram P Kandarpa V Rajendram M. The impact and applications of social media platforms for public health responses before and during the COVID-19 pandemic: systematic literature review. J Med Internet Res. (2022) 24:e33680. 10.2196/33680 35129456 PMC9004624

[28] Teoh JY Cacciamani GE. Gomez Rivas J. Social media and misinformation in urology: what can be done? BJU Int. (2021) 128:397. 10.1111/bju.15517 34581477

