# Supplementary material for: TikTok and YouTube as sources of information on anal fissure: A comparative analysis
Source: Front Public Health. 2022 Nov 3;10:1000338. doi: 10.3389/fpubh.2022.1000338 (PMC9669434; doi:10.3389/fpubh.2022.1000338)
Supplement: Supplementary file 1 [file Table_1.DOCX]

***Supplementary Material***

**Table 1. Average score per DISCERN question among all included TikTok/YouTube videos**

|  | Question | Average score (TikTok/YouTube) |
| --- | --- | --- |
| Section 1 | | |
| 1 | Are the aims clear? | 3.23/4.18 |
| 2 | Does it achieve its aims? | 3.32/4.09 |
| 3 | Is it relevant? | 3.68/4.39 |
| 4 | Is it clear what sources of information were used to compile the publication (other than the author or producer)? | 1.00/1.31 |
| 5 | Is it clear when the information used or reported in the publication was produced? | 1.00/1.31 |
| 6 | Is it balanced and unbiased? | 3.28/3.10 |
| 7 | Does it provide details of additional sources of support and information? | 1.02/1.78 |
| 8 | Does it refer to areas of uncertainty? | 1.15/1.71 |
| Section 2 | | |
| 9 | Does it describe how each treatment works? | 2.04/2.12 |
| 10 | Does it describe the benefits of each treatment? | 2.20/2.04 |
| 11 | Does it describe the risks of each treatment? | 1.28/1.94 |
| 12 | Does it describe what would happen if no treatment is used? | 1.58/1.90 |
| 13 | Does it describe how the treatment choices affect overall quality of life? | 2.03/1.92 |
| 14 | Is it clear that there may be more than 1 possible treatment choice? | 2.22/3.39 |
| 15 | Does it provide support for shared decision making? | 2.35/2.52 |
| Section 3 | | |
| 16 | Based on the answers to all of these questions, rate the publication’s overall quality as a source of information about treatment choices. | 2.56/2.45 |

**Table 2. Global Quality scale (GQS)**

|  | Description | Average score (TikTok/YouTube) |
| --- | --- | --- |
| Score 1 | Videos have poor flow, poor quality of the video, most information missing and are not at all useful for patients. | 2.93/2.92 |
| Score 2 | Videos have generally poor quality, some information listed and are of very limited use to patients. |  |
| Score 3 | Videos have moderate quality, and some important information is adequately discussed. |  |
| Score 4 | Videos have good flow, good quality, and most of the relevant information is listed and useful for patients. |  |
| Score 5 | Videos have excellent flow and quality and are very useful for patients. |  |
